# Enhanced Passive GNSS-Based Radar Imaging Based on Coherent Integrated Multi-Satellite Signals

**DOI:** 10.3390/s20030842

**Published:** 2020-02-04

**Authors:** Yu Zheng, Zhuxian Zhang, Lu Feng, Peidong Zhu, Feng Zhou

**Affiliations:** 1 College of Electronic Communication and Electrical Engineering, Changsha University, Hongshan Road # 98, Changsha 410022, China; zpd@ccsu.edu.cn (P.Z.); zhoufengcs@csu.edu.cn (F.Z.); 2 College of Electronic Science, National University of Defense Technology, Changsha 410073, China; zxzhang@ccsu.edu.cn (Z.Z.); Z20090838@ccsu.edu.cn (L.F.); 3 College of Computer Science and Technology, National University of Defense Technology, Changsha 410073, China

**Keywords:** GNSS radar, imaging gain, computational complexity, coherently integrated multi-satellites

## Abstract

Weak reflected signal is one of the main problems in a recent developing remote sensing tool—passive GNSS-based radar (GNSS radar). To address this issue, an enhanced GNSS radar imaging scheme on the basis of coherently integrating multiple satellites is proposed. In the proposed scheme, to avoid direct signal interference at surveillance antenna, the satellites that used as transmission of opportunity are in backscattering geometry model. To coherently accumulate echo signal magnitudes of the scene center in the targeted sensing region illuminated by the selected satellites, after performing the paralleled range compressions, a coordinates alignment operator is performed to the respective range domains, in which, pseudorandom noise (PRN) code phases are aligned. Thereafter, the coordinates aligned range compressed signals of the selected satellites are coherently integrated along azimuth domain so that imaging gain is improved and azimuth processing can be accomplished in only one state operation. The theoretical analysis and field proof-of-concept experimental results indicate that compared to both conventional bistatic imaging scheme and the state-of-the-art multi-image fusion scheme, the proposed scheme can provide a higher imaging gain; compared to the state-of-the-art multi-image fusion scheme, the proposed scheme has a less computational complexity and faster algorithm speed.

## 1. Introduction

Global Navigation Satellite System (GNSS) is a satellite system that produces and transmits radio signals for navigation and positioning purposes at global coverage level [1,2]. Up to the year 2020, modernization restructuring of the main GNSS systems, i.e., GPS, GLONASS, Galileo, and Compass (Beidou), will be completed. Throughout existing research works in GNSS area, there already has a wide range of surveys with respect to improving navigation and positioning accuracy on the basis of direct signals. However, in the last decade, the utility of multi-path GNSS signal, known as GNSS reflected signal, gained much attention. Passive GNSS-based radar (GNSS radar) [3,4] is a typical system that uses the reflected GNSS signal as source of opportunity for environmental surveillance. Compared to traditional active radar [5], as there is no need to construct a radar transmission platform, GNSS radar has a lower cost budget and is more flexible for installation under many environmental sensing scenarios. Compared to other passive radar, such as DVT-B based radar [6], because GNSS signals are global coverage and the transmission never failed, GNSS radar can perform all day all weather surveillance.

GNSS radar technique originates from GNSS Reflectometry (GNSS-R). The conventional GNSS-R technique contains two modes—non-geometric mode and geometric mode. Non-geometric mode is carried out based on received reflected signal strength or signal-to-noise ratio (SNR), whereas geometric mode is carried out based on the correlator output between reflected and direct signals [7]. As spatial information of targets cannot be provided by GNSS-R, recently, the technique is further developed into GNSS radar imaging. For stationary object, generally GNSS-synthetic aperture radar (GNSS-SAR) image is formatted, whereas for moving target identification, generally a Range Doppler (RD) map is formed. Based on the GNSS radar platform, at first, direct signal is synchronized for generating imaging matched filer signal. Then, conventional bistatic GNSS-SAR image formation stage contains two separated compressions, i.e., range compression and azimuth compression [8]. In terms of bistatic GNSS radar RD map formation, the only difference is that azimuth domain is processed by Fourier Transform (FT) [9]. On the basis of GNSS-SAR image formation scheme and GNSS radar RD map forming scheme, the feasibility of GNSS radar imaging was demonstrated under many environmental scenarios [9,10,11,12,13,14,15]. Meanwhile, in recent years, multi-static radar scheme was used for image formation in [9,13,16,17,18,19] based on multiple bistatic images fusion, which is considered as state-of-the-art imaging scheme in the relevant researches for GNSS radars.

Additionally, resolution of GNSS radar is investigated in the literatures [20,21,22,23,24,25]. For instance, the authors of [22] employed joint Galileo E5 signals to achieve range resolution at 3 meter level. Meanwhile, to separate multiple targets within one pseudorandom noise (PRN) code length, the first author’s previous works [24,25] improved range resolution based on the reflected signals at intermediate frequency (IF) level and performed secondary order differentiation operator on range compressed signals, respectively. In terms of azimuth resolution for GNSS-SAR, it mainly depends on the receiver moving trace and dwell time. For example, the authors of [20,23] improved azimuth resolution by fusion the bistatic images from different azimuth angles. The authors of [21] showed that using the dwell time 5 min, the azimuth resolution can be obtained at the level of 3–4 meters.

At the same time, there has the implementation based works with respect to GNSS radar. For instance, the authors of [26,27,28] demonstrated the applicability of GNSS-SAR for surface change detection based on carrier phase of reflected GNSS signals or correlation coefficient of two GNSS-SAR images obtained at the same time slot of different days. The work [29] implemented GPS signals for ocean surveillance, in which, the respective backscattering property of reflected signals is investigated. The authors of [30] investigated the applicability of aircraft detection using passive GNSS-based radar.

Among the existing GNSS radar related works, weak reflected signal remains one of the essential problems. As the distance between GNSS satellites and earth surface is approximately 22,200 km, the power flux density of direct signal at ground can be as low as −120 dBm to −130 dBm. The situation can become even worse for reflected signal. With such low power density, in many cases, a noisy bistatic GNSS-SAR image or a noisy bistatic GNSS radar RD map occurs. Although multi-image fusion scheme based on different satellites [9,13,16,18,19] can bring some enhancement in image SNR, as multiple full preliminary GNSS radar images are required, it will add a large amount of computational burden for the system. Meanwhile, multi-image fusion is a noncoherent integration scheme, and the respective imaging gain is still lower compared to coherent integration.

This paper proposes a new GNSS radar imaging scheme that uses coherently integrated multiple satellites signals. In the proposed scheme, the satellites in the backscattering geometric mode are selected as source of opportunity. Based on the individual range compressed result of each selected satellite, one is used as a benchmark, then range coordinates alignment operator is generated and performed to align range axis of other satellites to the benchmark. Thereafter, the aligned range compressed signals are coherently accumulated for performing azimuth compression or azimuth FT, where the signal magnitude of the targeted scene center are coherently accumulated and azimuth processing can be accomplished in one state operation. Both theoretical analysis and proof of concept field experiments under land and ocean surveillance scenarios reveal that the proposed imaging scheme can provide a higher imaging gain and lower computational complexity compared to the state-of-the-art multi-image fusion scheme.

This paper is organized as follows. The considered geometry and signal model is given in Section 2. Section 3 analyzed imaging gain and computational complexity of the conventional bistatic imaging scheme and state-of-the-art multi-image fusion scheme, whereas the respective analysis for the proposed imaging scheme is provided in Section 4. Field experimental confirmation of the proposed imaging scheme is shown in Section 5. Section 6 discusses the associated problems in this research and the respective future improvements. Section 7 concludes the whole paper.

## 2. The Considered Geometry and Signal Model

**Figure 1:**
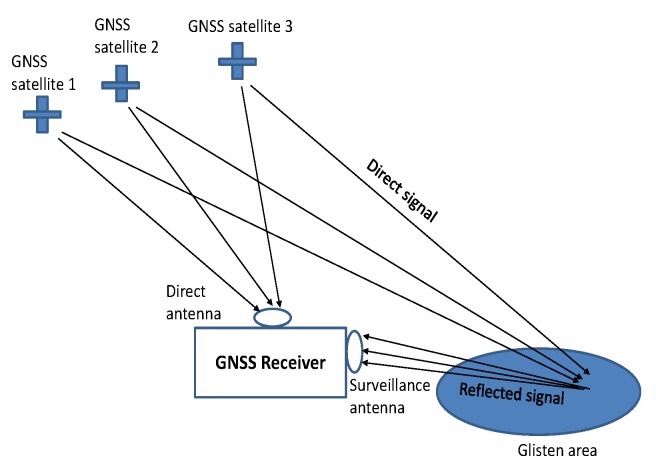
The considered geometry.

In this research, two separated antennas, i.e., direct antenna and surveillance antenna, are used for collecting direct and reflected GNSS signals, respectively. To avoid direct signal interference at surveillance channel for GNSS radar imaging, similar to [29], the satellites in the geometric position of backscattering are chosen as sources of opportunity. The geometry model is shown in Figure 1.

In Figure 1, for the ease of calibrating range migration when receiver is moving within certain trace for forming SAR image, both direct and surveillance antennas are mounted on the same platform. The signals received at direct antenna are used for synchronization, whereas the signals collected by surveillance antenna are used for radar imaging. At GNSS receiver, the received signals are digitized, downconverted to base-band and formed into range and azimuth domains. The respective mathematical model with respect to received direct signal for each satellite is given as

(1) $$\begin{array}{cc} s_{d}^{i}\left(t,u\right) & =A_{d}^{i}\cdot C\left(t-\tau^{i}\left(u\right)\right)\cdot D\left(t-\tau^{i}\left(u\right)\right)\\ \; & \;\cdot \exp\left(j\left(2\pi \omega_{d}^{i}\left(u\right)t+\phi_{d}^{i}\left(u\right)\right)\right)+n_{d}. \end{array}$$

where *i* represents the index for each satellite; $A_{d}^{i}$ represents the signal magnitude; *C* presents PRN code; *D* represents navigation message; *t* represents range domain, which is upper bounded by the length of PRN code; *u* represents azimuth domain, which is upper bounded by data collection duration or receiver moving duration; $\tau^{i}$ presents the transmission delay between receiver and each satellite; $\omega_{d}^{i}$ represents Doppler frequency of each satellite; $\phi_{d}^{i}$ represents carrier phase of each satellite; and $n_{d}$ represents noise at direct antenna. The parameters $\omega_{d}^{i}$ and $\phi_{d}^{i}$ can be considered as constants in the same range domain.

Each reflected signal can be considered as delayed version of the respective direct signal, which can be expressed as Equation (2)

(2) $$s_{r}^{i}\left(t,u\right)=\left\{\begin{array}{cc} A_{r_{i}}^{k}\cdot C\left(t-\tau^{i}\left(u\right)-\tau_{r_{i}}^{k}\left(u\right)\right)\cdot D\left(t-\tau^{i}\left(u\right)\right)\\ \cdot \exp\left(j\left(2\pi \omega_{r}^{i}\left(u\right)t+\phi_{r}^{i}\left(u\right)\right)\right)+n_{r} & \mathrm{presence}\;\mathrm{of}\;\mathrm{reflected}\;\mathrm{signal}\\ \;\\ n_{r} & \mathrm{absence}\;\mathrm{of}\;\mathrm{reflected}\;\mathrm{signal} \end{array}\right.$$

where *k* represents the index of each reflected signal at range domain, $\tau_{r_{i}}^{k}$ represents the reflected signal delay compared to the respective direct signal each satellite, $\omega_{r}^{i}$ represents Doppler frequency of reflected signal, $\phi_{r}^{i}$ represents carrier phase of reflected signal, and $n_{r}$ represents background noise at surveillance antenna. For stationary target, $\omega_{r}^{i}=\omega_{d}^{i}$, whereas for moving target, $\omega_{r}^{i}-\omega_{d}^{i}$ represents Doppler frequency caused by the object velocity. The parameters $\omega_{r}^{i}$ and $\phi_{r}^{i}$ can be considered as constants with the same range domain as well.

## 3. Analysis of Conventional Bi-Static Imaging Scheme and State of Art Multi-Images Fusion Scheme

GNSS radar is a passive radar, the transmitter and receiver should be located on separated platforms, thus only bistatic imaging scheme and multi-static imaging scheme are appropriate for such kind of system. In the GNSS radar-related research, bistatic imaging scheme [8] is regarded as a conventional scheme, which consists of the stages signal synchronization and radar imaging for individual satellite. Multi-static imaging scheme [9,13,16,18,19], known as the multi-image fusion scheme, is the state-of-the-art scheme, which is functions primarily on the basis of fusing multiple bistatic GNSS radar images. The detailed analysis of these two schemes are given as follows.

First, direct signal synchronization is carried out by tracking the received direct signal (1) from all the visible satellites. Using decoded navigation message, the satellite that satisfy the backscattering geometric model as Figure 1 is selected. In bistatic imaging, only one satellite that satisfied the geometric model in Figure 1 is selected as transmission of opportunity. Based on tracked code delay, carrier phase, and navigation bits for the selected satellite, the local replica is generated as

(3) $$\begin{array}{cc} s_{m}^{i}\left(t,u\right) & =C\left(t-\tau^{i}\left(u\right)\right)\cdot D\left(t-\tau^{i}\left(u\right)\right)\\ \; & \;\cdot \exp\left(j\left(2\pi \omega_{d}^{i}\left(u\right)t+\phi_{d}^{i}\left(u\right)\right)\right). \end{array}$$

Thereafter, for imaging stage, range compression is performed by correlating $s_{r}^{i}$ with $s_{m}^{i}$ per range domain along azimuth. The range compressed signal can be expressed as

(4) $$\begin{array}{cc} \; & R_{c}^{i}\left(2N_{s},u\right)\\ \; & \;=\frac{1}{N_{s}}\sum_{l_{r}=0}^{N_{s}-1}s_{r}^{i}\left(t,u\right)\cdot {\left(s_{m}^{i}\left(t-l_{r},u\right)\right)}^{*}\\ \; & \;=A_{r_{i}}^{k}\left(l_{u}\right)\cdot \Lambda \left(t-\tau^{i}\left(u\right)-\tau_{r_{i}}^{k}\left(u\right)\right)\cdot D\left(t-\tau^{i}\left(u\right)\right)\\ \; & \;\;\cdot \exp\left(j\left(2\pi \left(\omega_{r}^{i}\left(u\right)-\omega_{d}^{i}\left(u\right)\right)t+\phi_{r}^{i}\left(u\right)-\phi_{d}^{i}\left(u\right)\right)\right)\\ \; & \;\;+n_{rc} \end{array}$$

where $l_{r}$ denotes the range samples used for compression, $N_{s}$ denotes the respective samples quantity, * represents conjugate operator, $n_{rc}=\frac{1}{N_{s}}\sum_{l_{r}=0}^{N_{s}-1}n_{r}\cdot {\left(s_{m}^{i}\left(t-l_{r},u\right)\right)}^{*}$, and $\phi_{r}^{i}\left(u\right)-\phi_{d}^{i}\left(u\right)$ represents the bistatic range compressed carrier phase of the *i*-th satellite. The value $2N_{s}$ in $R_{c}^{i}$ is due to the fact that the quantity of samples is doubled after performing correlation operator.

For stationary object imaging, generally SAR image is formed. For GNSS-SAR image formation, azimuth matched filter is obtained on the basis of the result in Equation (4) along azimuth domain *u*. Azimuth compression is identically azimuth matched filtering, which can be expressed as

(5) $$T_{S}^{i}\left(2N_{s},2M_{u}\right)=\frac{1}{M_{u}}\sum_{l_{u}=0}^{M_{u}-1}R_{c}^{i}\left(2N_{s},u\right)\cdot {\left(R_{c}^{i}\left(2N_{s},u-l_{u}\right)\right)}^{*}$$

where $l_{u}$ represents azimuth samples for compression and $M_{u}$ denotes respective azimuth samples quantity. The value $2M_{u}$ in $T_{S}^{i}$ occurs because of the impact of azimuth correlator as well. For moving object, normally RD map is generated. The respective azimuth processing is carried out by performing FT of Equation (4) along azimuth domain, which can be expressed as

(6) $$T_{R}^{i}\left(2N_{s},M_{u}\right)=\frac{1}{M_{u}}\sum_{l_{u}=0}^{M_{u}-1}R_{c}^{i}\left(2N_{s},u\right)\cdot \exp\left(-j2\pi \omega l_{u}\right)$$

where ω represents azimuth frequency caused by the movement of object. To obtain GNSS-SAR image or GNSS radar RD map, coordinates in both range and azimuth domains are transformed into distance domains, and absolute operator $\left|\cdot \right|$ is applied on Equation (5) or Equation (6).

As for the state-of-the-art multi-image fusion scheme, more than one satellite are used for imaging. First, multiple bistatic images on the basis of each satellite are generated. Then, to transform the coordinates into distance domains, pseudo-range and elevating angle of each satellites are obtained on the basis of decoded navigation message. Thereafter, the scaling factor for image alignment is generated on the basis of the coordinates transformed results, which can be seen in detail in [19]. Assuming the scaling factor for performing alignment for each satellite is $\zeta^{i}$, the generation of GNSS-SAR image and GNSS radar RD map on the basis of multi-image fusion scheme can be expressed as Equations (7) and (8), respectively,

(7) $$T_{\mathrm{SAR}}=\frac{1}{m}\sum_{i=0}^{m-1}\left|T_{S}^{i}\left(2N_{s}\cdot \zeta^{i},M_{u}\right)\right|$$

(8) $$T_{\mathrm{RDM}}=\frac{1}{m}\sum_{i=0}^{m-1}\left|T_{R}^{i}\left(2N_{s}\cdot \zeta^{i},M_{u}\right)\right|$$

where *m* represents the quantity of satellites used for multi-image fusion.

We investigate imaging gain for conventional bistatic imaging and multi-image fusion schemes. In bistatic GNSS-SAR imaging scheme, after performing Equation (4), the range compression gain for individual satellite can be derived as $G_{rc}=N_{s}$; For azimuth compression Equation (5), the gain in the total dwell time can be derived as $G_{total}={\left(N_{s}\right)}^{2}\cdot M_{u}$; thus, the gain for azimuth compression can be expressed as $G_{ac}=\frac{{\left(N_{s}\right)}^{2}\cdot M_{u}}{N_{s}}=N_{s}\cdot M_{u}$. Therefore, the imaging gain for bistatic GNSS-SAR imaging scheme can be expressed as

(9) $$G_{imaging\_SAR}=G_{rc}\cdot G_{ac}={\left(N_{s}\right)}^{2}\cdot M_{u}$$

which is the same as $G_{total}$. For bistatic GNSS radar RD map formation, the gain in range compression is the same as bistatic GNSS-SAR imaging scheme. In terms of azimuth FT, the respective gain can be derived as $G_{af}=M_{u}$. Thus, the imaging gain can be expressed as

(10) $$G_{imaging\_RDM}=G_{rc}\cdot G_{af}=N_{s}\cdot M_{u}.$$

In terms of the multi-image fusion scheme, it can be regarded as the non-coherent integration of Equation (5) or (6) with coordinates alignment. Therefore, it can be easily derived that for multi GNSS-SAR images fusion, the gain is

(11) $$G_{multi\_SAR}=\sqrt{m}\cdot G_{imaging\_SAR}=\sqrt{m}\cdot {\left(N_{s}\right)}^{2}\cdot M_{u}.$$

For multi GNSS radar RD maps fusion, the gain is

(12) $$G_{multi\_RDM}=\sqrt{m}\cdot G_{imaging\_RDM}=\sqrt{m}\cdot N_{s}\cdot M_{u}.$$

From Equation (9) to (12), it can be seen that compared to bistatic imaging scheme, the gained strength for multi-image fusion is $\sqrt{m}$ larger.

Computational complexity in this paper is studied on the basis of number of operations. In signal synchronization stage of both bistatic imaging scheme and multi-image fusion scheme, as all the satellites are used for processing, the complexities are the same. Thus, only the complexity during imaging stage is considered. In terms of bistatic imaging, for local replica generation, the number of operations in code modulation and carrier modulation is the same as $N_{s}\times M_{u}$, thus the respective complexity is derived as $O\left(2N_{s}\times M_{u}\right)$. For range compression state, there exists number of multiplications $N_{s}^{2}\times M_{u}$ and number of additions $N_{s}\times M_{u}$, thus the respective complexity is $O\left(N_{s}\left(N_{s}+1\right)\times M_{u}\right)$. For azimuth compression for stationary object imaging, as the quantity of samples at range domain is doubled after performing compression, the number of multiplications is derived as $M_{u}^{2}\times 2N_{s}$ and the number of additions is derived as $M_{u}\times 2N_{s}$; thus, the respective complexity is derived as $O\left(M_{u}\left(M_{u}+1\right)\times 2N_{s}\right)$. Thereafter, the accumulated complexity for bistatic GNSS-SAR imaging stage can be derived as

(13) $$O\left(N_{s}\times M_{u}\times \left(5+N_{s}+2M_{u}\right)\right).$$

As for bistatic RD map generation for moving object detection, the complexity for local replica generation and range compression is the same as bistatic GNSS-SAR imaging. However, in the azimuth FT state, there exists number of multiplications $2N_{s}\times \frac{M_{u}}{2}\log_{2}M_{u}$ and number of additions $2N_{s}\times M_{u}\log_{2}M_{u}$. Thus, the respective complexity is derived as $O\left(3N_{s}\times M_{u}\log_{2}M_{u}\right)$. Therefore, the accumulated complexity for RD map generation stage can be derived as

(14) $$O\left(N_{s}\times M_{u}\times \left(3+N_{s}+3\log_{2}M_{u}\right)\right).$$

For multi-image fusion scheme, through a similar theoretical analysis, the number of operations for local replica generation, range compression, azimuth compression for stationary object detection, and azimuth FT for moving object indication can be derived as $2N_{s}\times M_{u}\times m$, $N_{s}\left(N_{s}+1\right)\times M_{u}\times m$, $M_{u}\left(M_{u}+1\right)\times 2N_{s}\times m$, and $3N_{s}\times M_{u}\times m\times \log_{2}M_{u}$, respectively. The number of operators for both coordinates alignment and images combination with respect to GNSS-SAR imaging can be derived as $2N_{s}\times 2M_{u}\times m$, whereas for RD map forming, the respective number of operations can be derived as $2N_{s}\times M_{u}\times m$. Therefore, the accumulated complexity for multi GNSS-SAR images fusion can be derived as

(15) $$O\left(N_{s}\times M_{u}\times m\times \left(11+N_{s}+2M_{u}\right)\right)$$

whereas for multi GNSS radar RD maps fusion, the complexity can be derived as

(16) $$O\left(N_{s}\times M_{u}\times m\times \left(7+N_{s}+3\log_{2}M_{u}\right)\right).$$

## 4. The Proposed Imaging Scheme Using Coherent Integrated GNSS Signals

To improve imaging gain and reduce computational burden than the state-of-the-art multi-image fusion scheme, a new imaging scheme on the basis of coherently integrating multiple GNSS satellites signals is proposed. The main principle of the proposed scheme is to coherently integrate coordinates aligned multi-satellites range compressed signals based on the compressed carrier phase difference. Thereafter, azimuth compression or azimuth FT can be performed by only once-through operation. The detailed analysis can be seen as follows.

The signal synchronization stage and range compression stage for the proposed imaging scheme is the same as multi-image fusion scheme. The criterion for selecting the multiple satellites as sources of opportunity should satisfy the model in Figure 1 as well. The initial range compressed signal without coordinates alignment is the same as Equation (4).

Thereafter, range coordinates alignment is performed. As it is hard to obtain the spatial information of the glisten region on the passive radar platform without generating full GNSS-SAR image or full GNSS radar RD map, unlike [19], the range coordinates alignment in the proposed scheme is carried out based on the synchronized carrier phase difference of direct signals among the selected satellites. The detailed derivation can be seen as follows.

**Figure 2:**
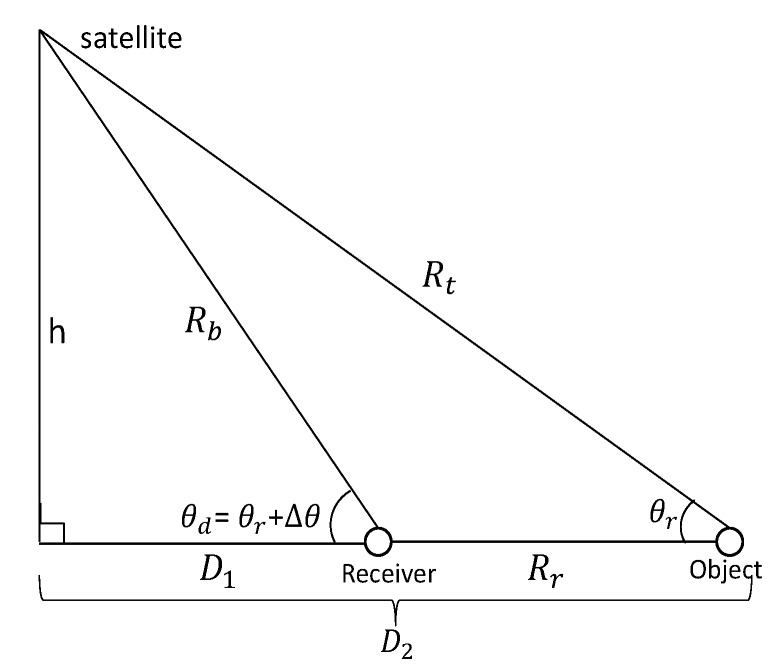
The schematic diagram of elevating angle.

**Figure 3:**
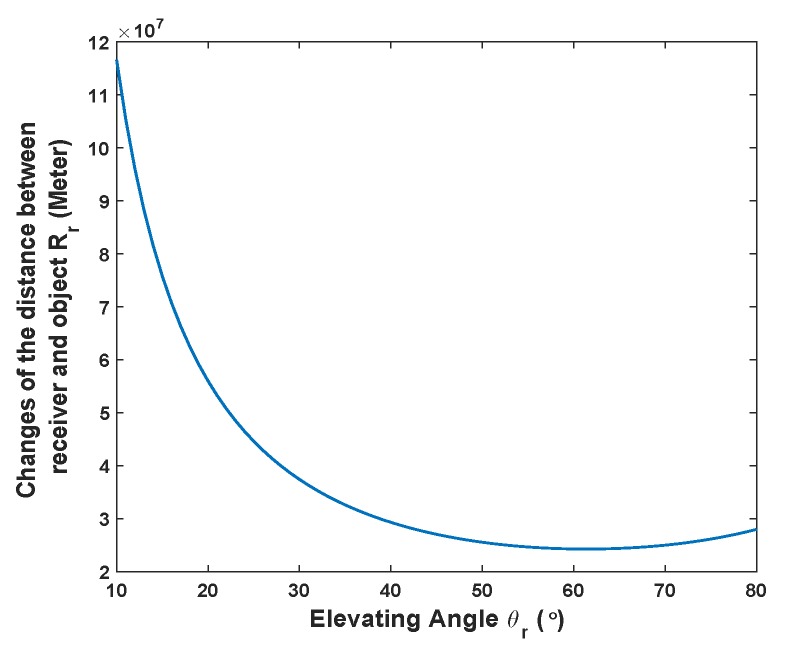
The relationship between $R_{r}$ and $\theta_{r}$.

- At first, bistatic range distance difference of the selected satellites for imaging is considered. We use one of the selected satellites as a benchmark and mark it as 0-th satellite. Assume, in the 0-th satellite, the distance between satellite and object is $R_{t}^{0}$ and between object and receiver is $R_{r}^{0}$; then, the respective bistatic range can be calculated as $d_{0}=R_{r}^{0}+R_{t}^{0}$. Assume for the satellite *i*, the distance between satellite and object is $R_{t}^{i}$, and between object and receiver is $R_{r}^{i}$; similarly, the respective bistatic range is calculated as $d_{i}=R_{r}^{i}+R_{t}^{i}$. Because the distance between object and receiver is not related to satellite position, we can have that $R_{r}^{i}=R_{r}^{0}$. Thus, the bistatic range difference is derived as
  (17) $$\begin{array}{cc} \Delta d_{i}\left(u\right) & =d_{i}\left(u\right)-d_{0}\left(u\right)\\ \; & =\left(R_{r}^{i}\left(u\right)+R_{t}^{i}\left(u\right)\right)-\left(R_{r}^{0}\left(u\right)+R_{t}^{0}\left(u\right)\right)\\ \; & =R_{t}^{i}\left(u\right)-R_{t}^{0}\left(u\right). \end{array}$$
  We investigate the impact in the changes of range distance between object and receiver with respect to GNSS satellite elevation angle. For the ease of analysis, we plot a respective schematic diagram in Figure 2.
  In Figure 2, $R_{b}$ represents the distance between satellite and receiver; *h* represents vertical distance between satellite and earth surface; $\theta_{d}$ and $\theta_{r}$ represent elevating angles at receiver and at object, respectively; and Δθ represents the difference between $\theta_{d}$ and $\theta_{r}$. Based on Figure 2, the changes of range distance between object and receiver $R_{r}$ with respect to $\theta_{r}$ and Δθ is calculated as
  (18) $$\begin{array}{cc} R_{r} & =D_{2}-D_{1}\\ \; & =\frac{h}{\tan\theta_{r}}-\frac{h}{\tan\left(\theta_{r}+\Delta \theta \right)}. \end{array}$$
  We study the changes of $R_{r}$ when Δθ reaches its minimum value $1^{\circ }$. Under the circumstance, GPS satellite is used as an example, in which the average vertical distance h= 22,200 km, the relationship between $R_{r}$ and $\theta_{r}$ is simulated in Figure 3, where the general interval of elevating angle for GNSS satellite is between $10^{\circ }$ and $80^{\circ }$ [31]. From Figure 3, note that to reach the level of elevation angle change by only $1^{\circ }$, the change of $R_{r}$ should be at the level more than $10^{7}$ meter. As GNSS radar is a passive radar, it is unlikely that the sensing range can reach that level. Therefore, under majority circumstance, it can be regarded that $\theta_{d}=\theta_{r}$. Then, the expression Equation (17) can be transformed into Equation (19) as follows.
  (19) $$\begin{array}{cc} \Delta d_{i}\left(u\right) & =R_{b}^{i}\left(u\right)-R_{b}^{0}\left(u\right)\\ \; & =\frac{f}{c}\left(\phi_{d}^{i}\left(u\right)-\phi_{d}^{0}\left(u\right)\right) \end{array}$$
  where $R_{b}^{i}$ and $R_{b}^{0}$ represent the base-line distance between receiver and the 0-th and the *i*-th satellite, respectively; *c* represents signal transmission speed; and *f* represents signal carrier frequency. The carrier phase values $\phi_{d}^{i}$ and $\phi_{d}^{0}$ of the *i*-th and 0-th satellites can be obtained from tracked results of Equation (1).
- Second, the necessity for performing coordinates alignment is evaluated. If $\Delta d_{i}<\frac{c}{N_{s}}\times 10^{-3}$, where $\frac{c}{N_{s}}\times 10^{-3}$ represents range distance between two sampling point within the period 1 ms of the PRN code for civil purpose, there is no need to perform coordinates transform; otherwise, perform coordinates alignment.
- Third, the aligned coordinates using the 0-th satellite as a benchmark can be derived as Equation (20)
  (20) $$\eta_{i}=\left\{\begin{array}{cc} \left(\frac{c}{N_{s}}\times 10^{-3}\right)\cdot l_{r}-\Delta d_{i}\left(u\right) & \mathrm{when}\mathrm{index}\mathrm{of}\mathrm{range}\mathrm{compressed}\mathrm{sample}>0\\ -\left(\frac{c}{N_{s}}\times 10^{-3}\right)\cdot l_{r}-\Delta d_{i}\left(u\right) & \mathrm{when}\mathrm{index}\mathrm{of}\mathrm{range}\mathrm{compressed}\mathrm{sample}<0 \end{array}\right.$$

After coordinates of the satellites are well aligned with the 0-th satellite, range compressed signals with respect to the satellites as illuminator of opportunity are accumulated coherently along azimuth, which can be expressed as

(21) $$R_{c}\left(\eta_{i},u\right)=\frac{1}{m}\sum_{i=0}^{m-1}R_{c}^{i}\left(\eta_{i},u\right).$$

After performing Equation (21), the scene center of the point spread function (PSF) with respect to illuminated region can be coherently accumulated. However, the illuminated ambiguity region of PSF with respect different satellites will be different. This will negatively impact on range resolution after performing Equation (21). As the main aim in this paper is the preliminary feasibility investigation with respect to imaging on the basis of coherently integrated multiple GNSS satellites, the range resolution problem is not specifically concentrated.

For stationary object imaging, azimuth compression is carried out based on the result Equation (21) along azimuth domain, which can be expressed as

(22) $$\begin{array}{cc} T_{s} & =\frac{1}{M_{u}}\sum_{l_{u}=0}^{M_{u}-1}R_{c}\left(\eta_{i},u\right)\cdot R_{c}^{*}\left(\eta_{i},u-l_{u}\right)\\ \; & =\frac{1}{M_{u}}\sum_{l_{u}=0}^{M_{u}-1}\left(\frac{1}{m}\sum_{i=0}^{m-1}R_{c}^{i}\left(\eta_{i},u\right)\right)\\ \; & \;\;\;\;\cdot {\left(\frac{1}{m}\sum_{i=0}^{m-1}R_{c}^{i}\left(\eta_{i},u-l_{u}\right)\right)}^{*}\\ \; & =\frac{1}{M_{u}m^{2}}\sum_{l_{u}=0}^{M_{u}-1}\left(\sum_{i=0}^{m-1}R_{c}^{i}\left(\eta_{i},u\right)\right)\\ \; & \;\;\;\;\cdot {\left(\sum_{i=0}^{m-1}R_{c}^{i}\left(\eta_{i},u-l_{u}\right)\right)}^{*}. \end{array}$$

For moving target detection, on the basis of Equation (21), azimuth FT is performed as follows.

(23) $$\begin{array}{cc} T_{RD} & =\frac{1}{M_{u}}\sum_{l_{u}=0}^{M_{u}-1}R_{c}\left(\eta_{i},u\right)\cdot \exp\left(-j\omega \cdot l_{u}\right)\\ \; & =\frac{1}{M_{u}\cdot m}\sum_{l_{u}=0}^{M_{u}-1}\left(\sum_{i=0}^{m-1}R_{c}^{i}\left(\eta_{i},u\right)\right)\\ \; & \;\;\;\;\;\cdot \exp\left(-j\omega \cdot l_{u}\right). \end{array}$$

Applying absolute operator on Equation (22) or (23), final GNSS-SAR image or GNSS radar RD map with respect to coherently integrated satellites can be obtained.

In summary, the work-flow of the proposed imaging scheme is given as Algorithm 1.
**Algorithm 1** The work-flow of the proposed imaging scheme1.Performing signal synchronization, selecting the GNSS satellites that satisfy the model as Figure 1. 2.On the basis of the selected satellites, generating local replica for each satellite as Equation (3). 3.Performing range compression for each selected satellite independently as Equation (4). 4.Using one of the selected satellite as a benchmark, mark it as 0-th satellite, extracting carrier phases of direct signals on the basis of the selected satellites. If $\Delta d_{i}=\frac{f}{c}\left(\phi_{d}^{i}-\phi_{d}^{0}\right)>\frac{c}{N_{s}}\times 10^{-3}$, performing coordinates alignment, otherwise directly jump to step 6. 5.Obtaining the coordinates alignment factor as Equation (19), and performing range coordinates alignment along azimuth as Equation (20). 6.Coherently combining the range compressed signals along azimuth direction of the selected satellites as Equation (21). 7.For stationary target imaging, performing azimuth compression as Equation (22). for moving target detection, performing azimuth FT as Equation (23). 8.Applying absolute operator on Equation (22) or (23), obtaining final GNSS-SAR image or GNSS radar RD map.

Imaging gain and computational complexity of the proposed imaging scheme are analyzed. The gained strength for each satellite in range correlation operation for the compression is the same as the bistatic imaging scheme. However, after performing Equation (21), the gain can be derived as $G_{r\_proposed}=N_{s}\cdot m$, after performing Equation (22), and the azimuth gain can be derived as $G_{a\_proposed}=\frac{N_{s}^{2}\cdot m^{2}\cdot M_{u}}{N_{s}}$. Thus, the gain for GNSS-SAR imaging under the proposed scheme can be derived as

(24) $$G_{SAR\_proposed}=G_{r\_proposed}\cdot G_{a\_proposed}=N_{s}^{2}\cdot m^{2}\cdot M_{u}.$$

After performing Equation (23), the gain is derived as $G_{af\_proposed}=M_{u}\cdot m$. Thus, the imaging gain for RD map generation under the proposed scheme can be derived as

(25) $$G_{RD\_proposed}=G_{r\_proposed}\cdot G_{af\_proposed}=N_{s}\cdot M_{u}\cdot m.$$

Comparing Equation (24) with Equation (11), it can be seen that the gained strength in GNSS-SAR imaging under the proposed scheme is $m^{\frac{3}{2}}$ higher than multi-image fusion scheme; comparing Equation (25) with Equation (12), it can be seen that the gained strength for GNSS radar RD map generation under the proposed scheme is $m^{\frac{1}{2}}$ higher than multi-image fusion scheme.

Computational complexity of the proposed scheme is investigated. Through the analysis of number of operations in each step of the proposed imaging scheme, we can have that the accumulated complexity for GNSS-SAR imaging under the proposed scheme is

(26) $$O\left(N_{s}\times M_{u}\times \left(\left(7+N_{s}\right)\times m+2\left(M_{u}+1\right)\right)\right)$$

whereas for GNSS radar RD map generation, it is

(27) $$O\left(N_{s}\times M_{u}\times \left(\left(7+N_{s}\right)\times m+3\log_{2}M_{u}\right)\right).$$

Comparing Equation (26) with Equation (15), it can be seen that the proposed scheme has less complexity by $2N_{s}\times M_{u}\times \left(2m+\left(M_{u}\left(m-1\right)-2\right)\right)$ number of operations than the multiple GNSS-SAR images fusion scheme; comparing Equation (27) with Equation (16), it can be seen that the proposed scheme has a less complexity by $3N_{s}\times M_{u}\times \left(m-1\right)\log_{2}M_{u}$ than multiple GNSS RD maps fusion scheme.

## 5. Proof-of-Concept Field Experimental Confirmation

**Figure 4:**
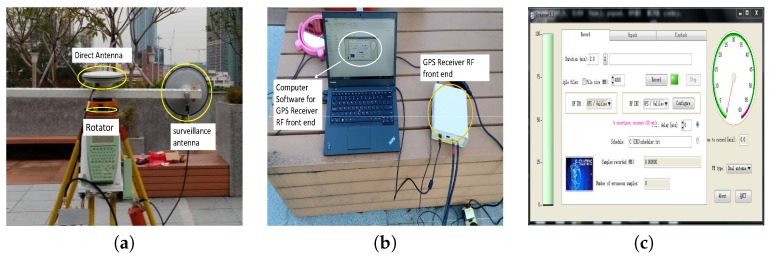
(**a**) The configuration of direct and surveillance antennas. (**b**) The software defined GPS receiver front end. (**c**) Interface of the computer software for the receiver front end data collection.

To validate the proposed scheme for enhancing GNSS radar imaging, field proof-of-concept experiments were carried out on the basis of software defined radio (SDR) GPS C/A code receiver. The same experimental equipment as first author’s previous work [32] is employed in this research. The equipments at receiver end are illustrated in Figure 4.

In Figure 4a, the direct antenna is right hand circular polarization (RHCP), which faces the sky to collect direct GPS signal for performing signal synchronization. The surveillance antenna is left hand circular polarization (LHCP), which used for collecting reflected GPS signals for the targeted surveillance region. For maintaining time synchronization, both direct and reflected signals are saved by the same radio frequency (RF) front end with two separated channels. The model of the RF front end is ET09/C, which produced by ip-solution company (http://www.ip-solutions.jp). The interface of the software for signal data collection is given in Figure 4c. The collected raw signal data were processed on commercial computer platform. The parameter values for the experiments is given in Table 1.

From Table 1, the range distance between two sampling points can be derived as follows.

(28) $$\begin{array}{cc} D_{s} & =\frac{c\cdot T}{N_{s}}\\ \; & =\frac{\left(3\times 10^{8}\mathrm{ms}\right)\times 1\mathrm{ms}}{16368}\\ \; & \approx 18m \end{array}$$

where *T* denotes PRN code period. Based on the experimental set-up and parameters, two representative scenarios, i.e., land imaging scenario with two strong reflectors and ocean target detection scenario, are considered. The detectability of the proposed scheme is examined based on imaging gains. The respective analyses can be seen as follows.

### 5.1. Land Imaging Scenario With Two Strong Reflectors

**Figure 5:**
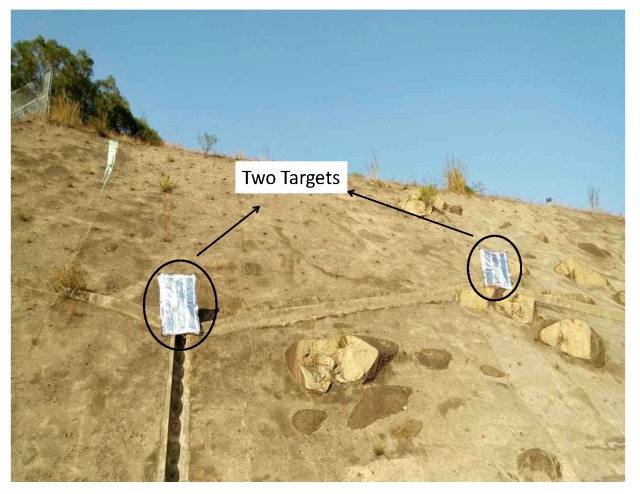
The optical image for land imaging scenario.

Under this scenario, GNSS radar worked in SAR imaging mode. The satellites were considered to be the stationary transmitters. The counterclockwise movement of the rotator in Figure 4a is employed for performing azimuth angular trace of surveillance antenna for aperture synthetic. The length of azimuth trace is $60^{\circ }$, while the moving duration is 1 min. Within the trace, optical image of the surveillance region is shown in Figure 5.

**Figure 6:**
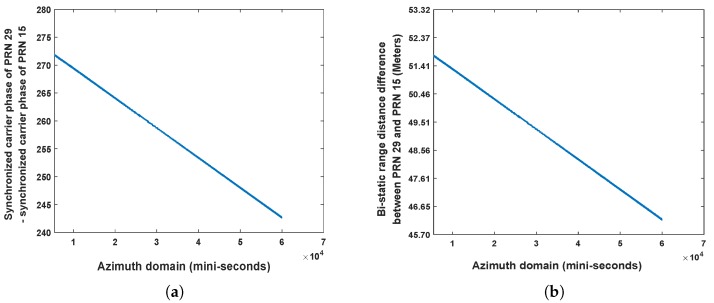
(**a**) Synchronized carrier phase of PRN 29 minus synchronized carrier phase of PRN 15. (**b**) Coordinates transformed results of panel (**a**) in distance domain.

In Figure 5, range distance between GNSS radar receiver and the slope is approximately 30–40 m, whereas the azimuth distance between the two reflector is ~$30^{\circ }$. The sizes of the two reflectors are 0.2m×0.2m. According to the decoded navigation message from direct signal synchronization, the satellites GPS PRN 15 and PRN 29 were used as source of opportunity, as they were only the satellites in the geometric position of backscattering under this scenario. First, the authors estimated bistatic range distance difference of the two satellites based on the carrier phase values from synchronization. The carrier phase difference can be seen in Figure 6a, whereas the coordinates transform results in distance domain can be seen in Figure 6b.

**Figure 7:**
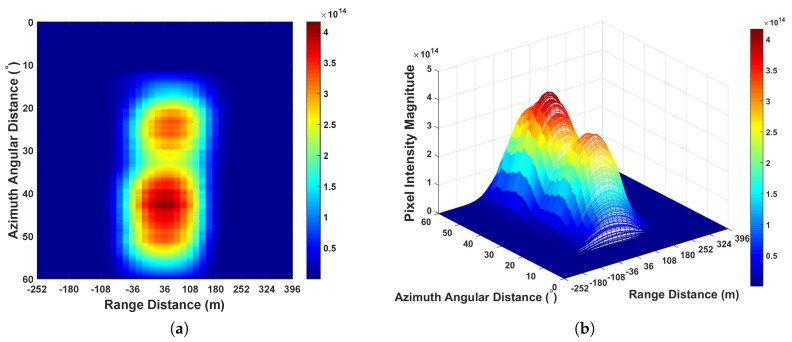
(**a**) The GNSS radar image obtained by the proposed imaging scheme. (**b**) Three-dimensional image of panel (**a**).

**Figure 8:**
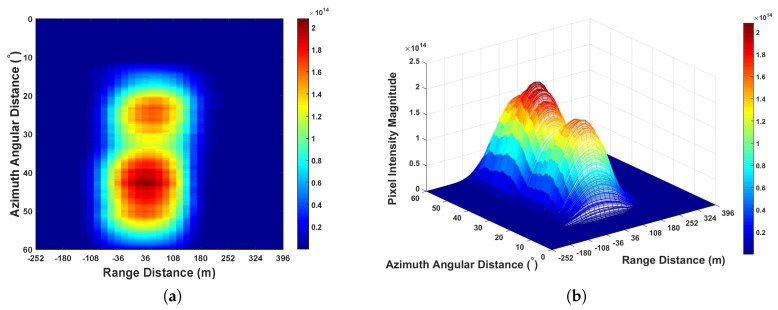
(**a**) The GNSS radar image obtained by multi-image fusion scheme. (**b**) Three-dimensional image of panel (**a**).

**Figure 9:**
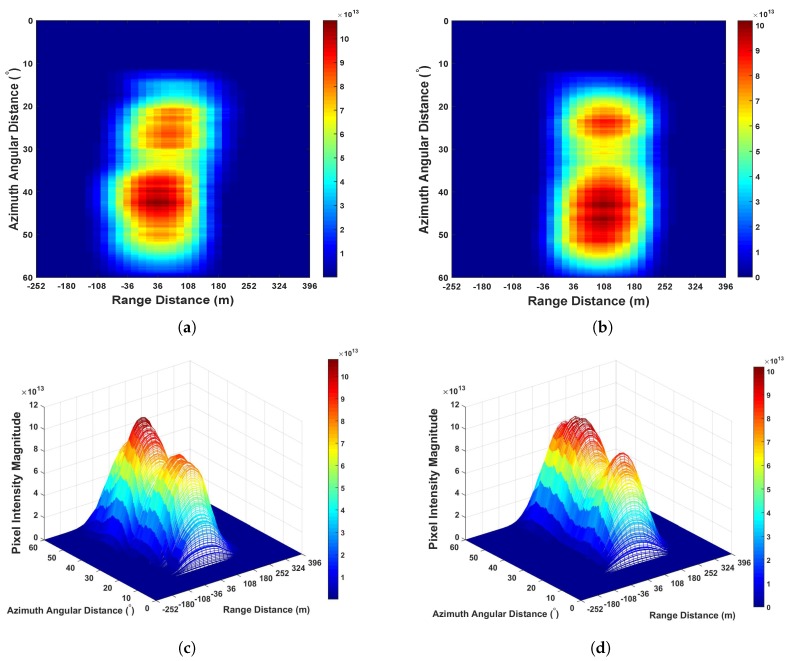
The GNSS bistatic SAR images: (**a**) The image obtained based on GPS PRN 15, (**b**) the image obtained based on GPS PRN 29, (**c**) three-dimensional image of panels (**a**), and (**d**)Three-dimensional image of panel (**b**).

From Figure 6, it can be seen that the error caused by the position of two satellites is larger than the range distance represented by the distance between two sampling points 18 m. Thus, it is required to perform range domain alignment. As PRN 29 is further than PRN 15, on the GNSS radar platform in this experiment, after performing the separated range compression stages, the authors used the bistatic range distance of PRN 15 as a benchmark, whereas the bistatic range distance of PRN 29 minus the values shown in Figure 6b per range domain along azimuth time for the calibration. Thereafter the range compressed signals of the two satellites are coherently integrated as Equation (21) for performing azimuth compression. The obtained GNSS radar image with respect to the proposed scheme can be seen in Figure 7. For comparison, the images obtained by multi-image fusion scheme and bistatic images of the two satellites are given in Figure 8 and Figure 9, respectively.

We investigated imaging gain by examining the highest pixel intensity. From Figure 7, Figure 8 and Figure 9, it can be seen that as the integration of bistatic results were employed, the imaging gain in both Figure 7 and Figure 8 are higher than Figure 9. Comparing Figure 7 to Figure 8, because the proposed imaging scheme works in the mode that coherently integrating the coordinates aligned range compressed signal, the imaging gain is approximately 2.83 higher than the state-of-the-art scheme multi-image fusion, where the value *m* in this scenario is 2.

The computational efficiency for this experiment is studied on the basis of numbers of operations during imaging and the respective algorithm speeds. From Table 1, we have that the number of samples in each range domain is 16368. As two satellites were used as signal of opportunity, the PRN code period is 1 ms and the moving duration for signal collection is 1 min, we can have that the number of samples in azimuth domain is $60\times 10^{3}$. Therefore, under the proposed imaging scheme, there exists $2\times 16368\times 60\times 10^{3}\times 2=3.93\times 10^{9}$ numbers of operations during local replica generation. For imaging range compression stage, the number of operations is $16368\times 16369\times 60\times 10^{3}\times 2=3.22\times 10^{13}$. The number of operations during coordinates alignment and range signal coherently accumulation are $3.93\times 10^{9}$ as well. As coordinate aligned range compressed signals are coherently integrated along azimuth, only one azimuth compression stage is needed. From the derivation with the experimental parameter values, the numbers of operations during one azimuth compression stage is $60\times 10^{3}\times \left(60\times 10^{3}+1\right)\times 2\times 16368=1.18\times 10^{14}$. Then, we can have that the accumulated number of operations during the procedure is approximated $1.20\times 10^{14}$. For multi-image fusion scheme, the difference under this scenario is that there exists two azimuth compression stages for the satellite GPS PRN 15 and PRN 29, respectively. Through a similar analysis, the number of operations during the imaging procedure can be derived as $2.02\times 10^{14}$. For bistatic imaging scheme with either GPS PRN 15 or PRN 29, as only one satellite is employed as transmitter of opportunity, the number of operations will be deducted by half during azimuth compression compared to multi-image fusion scheme, and deducted by half during local replica generation state and range compression state, compared to both the proposed imaging scheme and multi-image fusion scheme as well. Meanwhile, there is no coordinate alignment step. The number of operations with respect to the three schemes during imaging stage are summarized in Table 2.

The algorithm speeds are studied on the basis of machine running time under the same computer environment, which are concluded in Table 3.

From Table 2 to Table 3, it can be seen that the proposed scheme has less computational complexity than multi-image fusion scheme. Meanwhile, the proposed scheme is faster than multi-image fusion scheme. However, as both the proposed scheme and multi-images scheme need to generate local replica and perform range compression of the signals from the two satellites independently, their computational complexity and the time spends on the procedure for imaging are higher than bistatic imaging scheme.

### 5.2. Ocean Moving Object Detection Scenario

**Figure 10:**
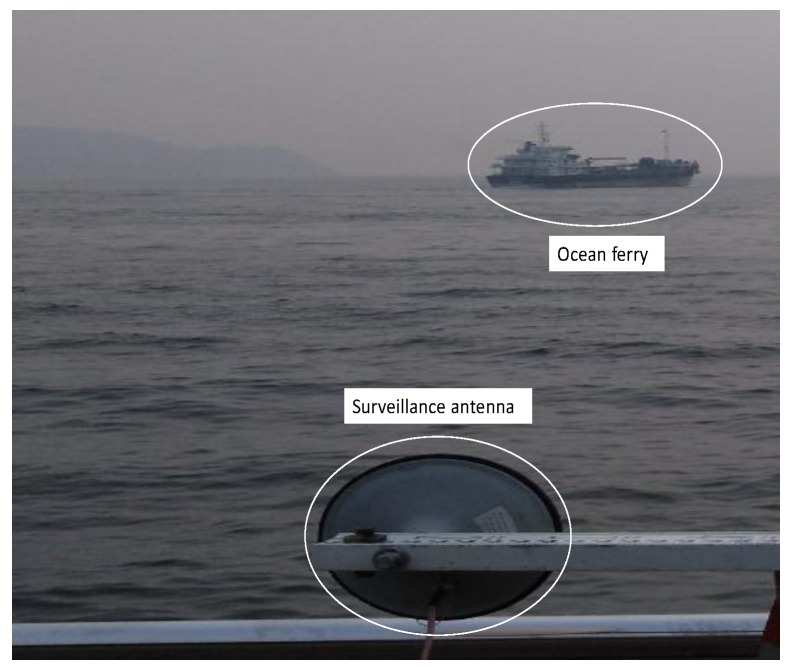
The optical image for ocean moving object surveillance scenario.

This subsection considers the ocean moving object surveillance scenario, in which RD maps are forming. The experiment was carried out at Hong Kong Cyberport. The optical image for this scenario is given in Figure 10.

**Figure 11:**
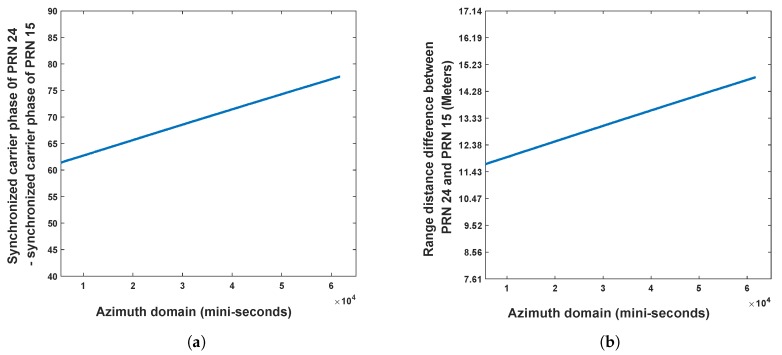
(**a**) Synchronized carrier phase of PRN 24 minus synchronized carrier phase of PRN 15. (**b**) Coordinates transformed result of panel (a) in range distance domain.

In this scenario, we aim to form RD maps of the ocean ferry. To form backscattering geometric model for imaging, on the basis of decoded navigation message, the satellites GPS PRN 15 and GPS PRN 24 are used as source of opportunity. From the measurement by laser range finder, the distance between the ferry and surveillance antenna is approximately 910–930 m. Both direct and surveillance antennas are fixed by the rotator (see Figure 4) for raw signal data collection. The duration for signal collection is 1 min. Therefore, the signals samples along azimuth domain is $60\times 10^{3}$ as well. Based on the respective signal collection set-up, first, the bistatic range distance difference along azimuth domain between the two satellites are extracted based on synchronized carrier phase value, which can be seen in Figure 11.

**Figure 12:**
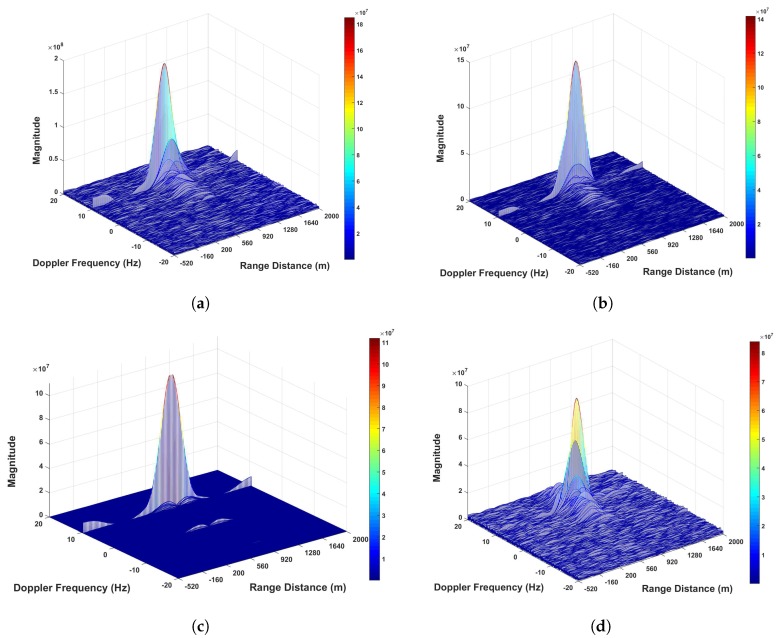
The GNSS radar RD maps for ocean surveillance: (**a**) The proposed imaging scheme. (**b**) Multi-images fusion scheme. (**c**) Bi-static imaging based on GPS PRN 15. (**d**) Bi-static imaging based on GPS PRN 24.

From Figure 11, it can be seen that the bistatic range distance difference between the two satellites is less than the distance between two sampling points 18 m. Thus, the respective range compressed signals can be directly integrated without performing coordinate alignment. After performing the coherent accumulation of the range compressed signals from the respective satellites, azimuth FT was carried out for obtaining RD maps. The generated RD maps based on the proposed imaging scheme, multi-image fusion scheme and bistatic imaging scheme are given in Figure 12. For the ease of comparison, the results are illustrated in three-dimensional mode.

Comparing the highest pixel intensity among each image in Figure 12, we can see that in the proposed imaging scheme, as the range signals are coherently integrated, indeed Figure 12a has the highest imaging gain. Because multi-image fusion scheme is a non-coherent integrated scheme, the imaging gain in Figure 12b is not as high as Figure 12a.

Similar as Section 5.1, the efficiency of this experiment is investigated based on numbers of operations and algorithm speeds extracted from machine running time. Based on the parameter values in Table 1 and the data collection duration, for the proposed imaging scheme, the number of operations with respect to local replica generation is the same as the respective results in Section 5.1. For the ease of comparison, range compression was carried out at frequency domain in this experiment. Through analysis, it has the number of operations $2\times \frac{3}{2}\times$ 16,368 $\times \log_{2}$ (16,368) = 687,380 for range FT and range inverse FT, respectively. The number of operations for range multiplication for the compression is 2× 16,368 = 32,736. There is no operations for coordinates transform, whereas the number of operations for coherently accumulating range compressed of the selected satellites is the same as the respective result in Section 5.1 as well. As range compressed signals of GPS PRN 15 and PRN 24 are accumulated, only one stage azimuth FT is necessary. The number of operations with respect to complex multiplication and addition during performing azimuth FT in each range position are $\frac{60\times 10^{3}}{2}\log_{2}\left(60\times 10^{3}\right)\approx 4.76\times 10^{5}$ and $60\times 10^{3}\cdot \log_{2}\left(60\times 10^{3}\right)\approx 9.52\times 10^{5}$, respectively. Therefore the accumulated number of operations during imaging processing for RD map forming is derived as 2559500. For multi-image fusion scheme, there exists two azimuth FT processing for GPS PRN 15 and PRN 24, respectively. Thus, there will exist additional number of operations 1428000. The number of operations with respect to the proposed imaging scheme, multi-image fusion scheme and bistatic RD mapping scheme during the imaging stage are summarized in Table 4.

The algorithm speeds based on machine running time are illustrated in Table 5.

From Table 4 and Table 5, the same verdict as Section 5.1 can be concluded: the proposed imaging scheme has less computational burden and faster than multi-image fusion scheme.

## 6. Discussion

In this paper, due to the fact that it mainly focuses on the feasibility testing with respect to the proposed imaging scheme based on integrating satellites coherently, only bistatic range is considered. Thus, only the information carrier phase difference is used for range coordinates alignment. However, to estimate the objects location more precisely, the parameter values that elevating angle and pseudo-range between each satellite and receiver are still required. Therefore, in future, the authors aim to improve range coordinates alignment stage in the proposed imaging scheme for obtaining the objects range position on GNSS radar image more precisely and with a lower computational complexity than the respective state in multi-image fusion scheme.

Meanwhile, the field experiments are carried out on the ground-based GNSS radar. Under many circumstances, only two satellites are satisfied backscattering geometric position as Figure 1. Thus, to maximumly avoid direct signal interference at surveillance channel, only two satellites are used for the coherent accumulation. In the future, the authors aim to perform experiments on airborne GNSS radar platform. As all the satellites are satisfied under the geometric model, as in Figure 1, all of them can be employed as transmitter of opportunity, in which, the advantages of the proposed imaging scheme will be more significant in field implementations. At the same time, as the PSFs around the scene center of the same target illuminated by different satellites are not the same, although the magnitude of scene center can be improved by coherently accumulating range compressed signals, the range resolution will be negatively impacted. Therefore, in future, the authors will further improve the proposed scheme by combining the range resolution enhanced mechanism in the first author’s previous work [25]. In this remit, a range resolution enhanced GNSS-SAR or GNSS radar RD map can be obtained on the basis of coherently integrated multi-satellites.

In addition, for this research, the authors only have GPS C/A code SDR receiver as shown in Figure 4 for field experimental testing. In the future, the authors will apply the proposed imaging scheme to GLONASS, Galileo, and Beidou (Compass) signal receivers, in which, the adaptability of the proposed scheme can be enhanced.

## 7. Conclusions

A new imaging scheme on the basis of coherently integrating multiple GNSS satellites is proposed in this paper. In the proposed scheme, the satellites that satisfy backscattering model is selected as sources of opportunity. Based on the synchronized carrier phases of the selected satellites, range coordinate alignments among satellites are performed after performing range compressions independently. Thereafter, the coordinate aligned range compressed signals are coherently accumulated along azimuth domain, in which azimuth compression can be completed in only once-through operation. Both theoretical analysis and field proof of concept experiments show that compared to both conventional bistatic imaging scheme and state-of-the-art multi-image fusion scheme, the proposed imaging scheme can have a higher imaging gain; compared to multi-image fusion scheme, the proposed imaging scheme has a lower computational complexity. In conclusion, the proposed scheme will be more suitable for GNSS radar image formation under weak reflected signal circumstance.

## Figures and Tables

**Table 1:** The parameter values for field experimental demonstration.

Parameters	Types or Values
Supported signal type	GPS C/A code signal
Operating frequency	1575.42 MHz (L1 band)
Signal transmission velocity *c*	$3\times 10^{8}$ m/s
Code period *T*	1 ms
Signal bandwidth *B*	1.023 MHz
Sampling frequency for	
RF front end	$1.6368\times 10^{7}\mathrm{Hz}$
Antenna gain + RF gain	20 dB
Boltzmann constant *k*	$1.38\times 10^{-23}$ J/K
Experimental temperature	300 K

**Table 2:** The number of operations for land imaging scenario.

The Proposed Scheme	Multi-Images Fusion Scheme	Bi-Static Imaging
$1.20\times 10^{14}$	$2.02\times 10^{14}$	$9.15\times 10^{13}$

**Table 3:** The algorithm speeds for land imaging scenario.

The Proposed Scheme	Multi-Images Fusion Scheme PRN 15	Bi-Static Imaging for PRN 29	Bi-Static Imaging for
13,853.217 s	21,781.140 s	9895.502 s	9775.131 s

**Table 4:** The number of operations for RD map generation under ocean moving object surveillance scenario.

The Proposed Scheme	Multi-Images Fusion Scheme	Bi-Static Imaging
2,559,500	3,987,500	1,755,800

**Table 5:** The algorithm speed for ocean moving object surveillance.

The Proposed Scheme	Multi-Images Fusion Scheme	Bi-Static Imaging for PRN 15	Bi-Static Imaging for PRN 24
4235.228 s	7094.136 s	3386.120 s	3371.203 s

## Abbreviations

The following abbreviations are used in this paper:

GNSS	Global Navigation Satellite System
SAR	Synthetic Aperture Radar
SNR	Signal-to-noise Ratio
RD	Range Doppler
SDR	Software Defined Radio
RHCP	Right Hand Circular Polarization
LHCP	Left Hand Circular Polarization
RF	Radio Frequency
PSF	Point Spread Function
